# Early Nitrogenase Ancestors Encompassed Novel Active Site Diversity

**DOI:** 10.1093/molbev/msac226

**Published:** 2022-10-19

**Authors:** Sarah L Schwartz, Amanda K Garcia, Betül Kaçar, Gregory P Fournier

**Affiliations:** Microbiology Graduate Program, Massachusetts Institute of Technology, Cambridge, MA; Department of Earth, Atmospheric, and Planetary Sciences, Massachusetts Institute of Technology, Cambridge, MA; Department of Bacteriology, University of Wisconsin-Madison, Madison, WI; Department of Bacteriology, University of Wisconsin-Madison, Madison, WI; Department of Earth, Atmospheric, and Planetary Sciences, Massachusetts Institute of Technology, Cambridge, MA

**Keywords:** ancestral sequence reconstruction, nitrogenase, Nif, early life

## Abstract

Ancestral sequence reconstruction (ASR) infers predicted ancestral states for sites within sequences and can constrain the functions and properties of ancestors of extant protein families. Here, we compare the likely sequences of inferred nitrogenase ancestors to extant nitrogenase sequence diversity. We show that the most-likely combinations of ancestral states for key substrate channel residues are not represented in extant sequence space, and rarely found within a more broadly defined physiochemical space—supporting that the earliest ancestors of extant nitrogenases likely had alternative substrate channel composition. These differences may indicate differing environmental selection pressures acting on nitrogenase substrate specificity in ancient environments. These results highlight ASR's potential as an in silico tool for developing hypotheses about ancestral enzyme functions, as well as improving hypothesis testing through more targeted in vitro and in vivo experiments.

## Introduction

Ancestral sequence reconstruction (ASR) is widely used for modeling sequence properties and functions of ancient enzyme variants. Maximum-likelihood reconstructions combine substitution models with aligned sequence data and phylogenetic information to calculate the residue likelihood of aligned sites for all internal nodes in a tree (Merkl and Sterner 2016; Selberg Gaucher and Liberles 2021). This approach enables exploration of targeted hypotheses about ancestral proteins in silico and is also an essential starting point for enzyme resurrection and in vitro or in vivo activity assays (Garcia and Kaçar 2019; Liberles et al. 2020). Because most of Earth's earliest organisms and metabolisms have no preserved record, deep-time evolution must be inferred using modern genomic and biochemical data (Barnabas et al.1982). ASR models protein “fossils,” providing biological data about ancestral enzymes and organisms to consider in context with environmental data, such as geochemical or atmospheric modeling (Thornton 2004; Hochberg and Thornton 2017). This process enables quantitative assessment of otherwise-elusive evolutionary hypotheses, making ASR essential for studying ancient, geochemically relevant enzyme families (Garcia and Kaçar 2019).

Nitrogen-fixing nitrogenases are the lynchpin of Earth's biological nitrogen cycle, and have been extensively studied for their role in global biogeochemistry (Raymond et al. 2004; Canfield Glazer and Falkowski 2010; Sickerman et al. 2019). Modern nitrogenases are categorized by their metal cofactors (Sickerman et al. 2019; Raymond et al. 2004; Garcia et al. 2020; North et al. 2020). All diazotrophs express molybdenum nitrogenase (Nif), with its iron-molybdenum cofactor (FeMoCo); some nitrogen fixers also contain alternative vanadium nitrogenase (Vnf) or iron-only nitrogenase (Anf; Boyd et al. 2011; Sickerman et al. 2019; Garcia et al. 2020). Phylogenetic analyses, molecular clock data, and cofactor-type-specific nitrogen isotope fractionations support the parsimonious inference that Nif is the oldest, ancestral form of nitrogenase. Recent studies suggest that the ancestor of this enzyme may date to the early Archaean eon (Boyd et al. 2011; Stüeken et al. 2015; Garcia et al. 2020; Parsons et al. 2021).

In addition to their unique capacity for dinitrogen (N_2_) fixation, modern nitrogenases can promiscuously reduce other linear, triple-bond-containing molecules at the FeMoCo active site (Fani et al. 2000; Seefeldt et al. 2013). It is possible that these off-target substrates could have played a larger role in early Earth systems. Several alternative substrates for nitrogenase enzymes have been identified, including carbon dioxide (CO_2_), carbon monoxide (CO), acetylene (C_2_H_2_), and hydrogen cyanide (HCN; Li et al. 1982; Conradson et al. 1989; Seefeldt et al. 2013; Stripp et al. 2022). It has been proposed that HCN promiscuity reflects “relic” chemistry of nitrogenases from highly reduced early environments, with HCN serving as a substrate or a target for detoxification (Silver and Postgate 1973; Fani et al. 2000; Pickett et al. 2004). HCN was produced and maintained on early Earth (Tian et al. 2011; Ferus et al. 2017; Todd and Öberg 2020), especially during the Archaean eon (Zahnle 1986; Tian et al. 2011), and is a prominent candidate feedstock molecule for abiogenesis, as it can be abiotically converted into several key nucleic and amino acids (Oró 1961; Oró and Kamat 1961; Ferris and Orgel 1966; Ferris et al. 1978; Miyakawa et al. 2002; LaRowe and Regnier 2008; Pearce and Pudritz 2015; Sutherland 2016). Given the age of the nitrogenase ancestor, no direct biological or biochemical data exist to support the presence or significance of HCN in early ecosystems. But ASR may be able to constrain such hypotheses for ancient biochemistry (Garcia and Kaçar 2019).

A higher affinity for alternative substrates in ancestral nitrogenases would likely require different amino acid residues in the substrate-binding part of the enzyme (the substrate channel), while preserving catalytic activity. As ASRs are inherently probabilistic, individual sites may be inferred to have several possible ancestral states with non-negligible probabilities. Variation across sampled extant diversity allows the combinations within likely ancestor sequences to explode, producing a far greater number of plausible sequence ancestors than can be experimentally tested. Here, we use ASR to reconstruct the amino acid identities of alignment sites directly interacting with the substrate binding channel in the common ancestor of the active site-containing nitrogenase subunit (*nif/vnf/anfD*). A library of plausible sequence ancestors for this collection of sites was generated, and their relative probabilities calculated. We applied a simple hypothesis-testing strategy: Are likely ancestral active site sequence states found within sampled extant nitrogenase diversity? We find that this is not always the case, permitting the possibility that early nitrogenase ancestors had substrate binding channels with properties distinct from modern enzymes.

## Results

**Fig. 1.**
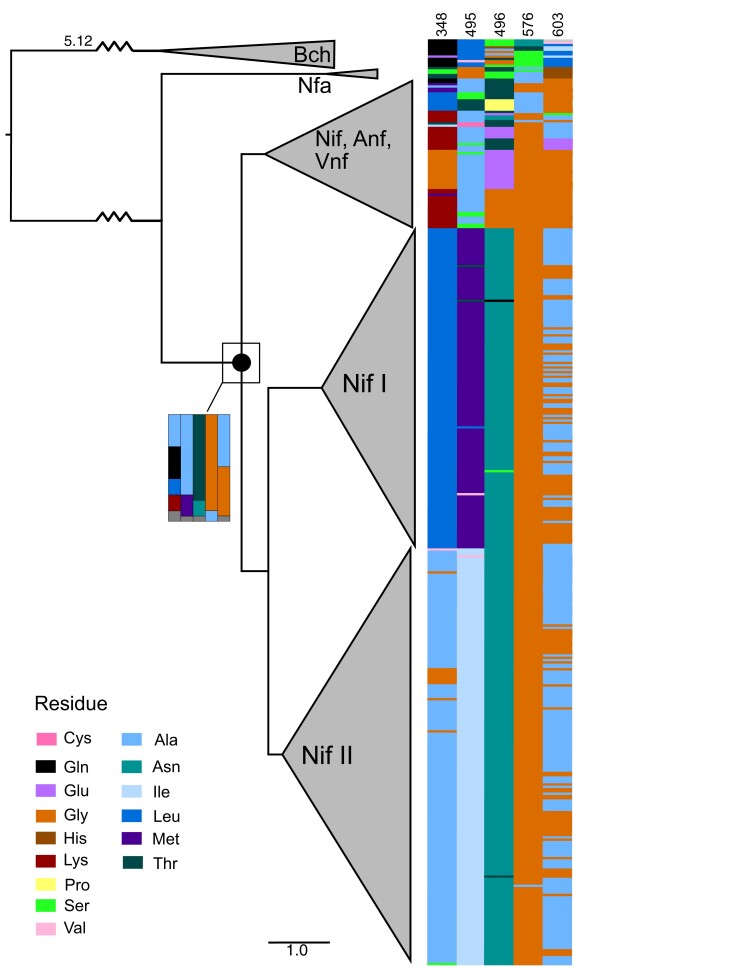
Extant nitrogenase sequence diversity. A maximum-likelihood gene tree for the D subunit of extant nitrogenases and outgroup sequences (Nfa, Group IV nitrogenase-like enzymes; Bch, bacteriochlorophyll/protochlorophyllide oxidoreductases), displaying the residue state at each of five variable sites in the *Azotobacter vinelandii nifD* substrate channel. The last common ancestor evaluated for nitrogenases is marked with a black node; reconstructed site likelihoods for all residues *P* > 0.10 are shown by residue color in the inset bar chart.

Of 24 sites known to play a role in substrate docking or coordination in modern *Azotobacter vinelandii* Nif (Seefeldt et al. 2013; Smith et al. 2014), five residues showed variability (>1 ancestral state with *P* > 0.10) in the predicted nitrogenase ancestor (table 1). For comparison with ASR results, sequence diversity for these five variable sites was evaluated in extant nitrogenases (fig. 1). Extant variation at these sites roughly maps onto the major nitrogenase groups in the gene tree. Sites 398 and 495 in the alignment highlight the distinction between Group I Nif (comprising well-studied proteobacterial and cyanobacterial *nifD* sequences, including that of *A. vinelandii*) and Group II Nif (comprising sequences from Clostridia, Bacteroidetes, and methanogenic archaea, among others; Raymond et al. 2004). Group I Nif conserves leucine (Leu) at site 395, whereas Group II conserves primarily alanine (Ala) (with fewer cases of glycine [Gly], and individual substitutions of serine [Ser] and cysteine [Cys]). Group I Nif contains primarily methionine (Met) at site 495, whereas Group II Nif primarily displays isoleucine (Ile). Variation at site 496, meanwhile, highlights the split between Nif I/II nitrogenases and the clade including alternative (Vnf, Anf) nitrogenases (which also contains several divergent Nif sequences): With very few exceptions, Group I and II nitrogenases conserve asparagine (Asn) at this site, whereas alternative clade sequences are poorly constrained, most frequently expressing glutamate (Glu), threonine (Thr), and Gly.

**Table 1.** ASR-Predicted Residue for key Substrate Channel Sites

Conserved residue in alignment (Av)	ASR Nif/Anf/Vnf ancestor residue, p(residue)	Other residues *P* > 0.10	Putative role in modern Nif active site	Corresponding residue in *Av nifD* (Seefeldt et al. 2013; Smith et al. 2014)
Val-182	Val, 0.99998	No	Modulates substrate access to catalytic core/active site	α-70^Val^
His-350	His, 0.99999	No	H-bond with FeMoCo, flexible (S2A or S2B)	α-195^His^
Tyr-497	Tyr, 0.99997	No	Substrate channel formation; gating residue	α-281^Tyr^
Arg-493	Arg, 0.99991	No	H-bond with FeMoCo in substrate channel (surface “flap” for substrate access)	α-277^Arg^
His-604	His, 1	No	Substrate channel formation; gating residue	α-383^His^
Cys-491	Cys, 1	No	Coordinates FeMoCo	α-275^Cys^
His-745	His, 0.99998	No	Coordinates FeMoCo	α-442^His^
Arg-224	Arg, 0.99912	No	Coordinates FeMoCo	α-96^Arg^
Gln-346	Gln, 0.99995	No	Coordinates FeMoCo	α-191^Gln^
Gly-181	Gly, 0.99934	No	Switch control for α-70^Val^ side chain	α-69^Gly^
Ser-347	Ser, 0.99888	No	N_2_ interaction in substrate channel	α-192^Ser^
Asn-155	Asn, 0.99972	No	Lines substrate channel	α-49^Asn^
Gly-178	Gly, 0.99994	No	Lines substrate channel	α-66^Gly^
Val-183	Val, 0.99824	No	Lines substrate channel	α-71^Val^
Ser-345	Ser, 0.99918	No	Lines substrate channel	α-190^Ser^
Leu-348	Ala, 0.29253	Gln, 0.27519; Leu, 0.16779; Lys, 0.16716	Lines substrate channel	α-193^Leu^
His-351	His, 0.99992	No	Lines substrate channel	α-196^His^
Asn-354	Asn, 0.99946	No	Lines substrate channel	α-199^Asn^
Ser-494	Ser, 0.99993	No	Lines substrate channel	α-278^Ser^
Met-495	Ala, 0.74242	Met, 0.17555	Lines substrate channel	α-279^Met^
Asn-496	Thr, 0.79331	Asn, 0.14693	Lines substrate channel	α-280^Asn^
Gly-576	Gly, 0.87529	Ala, 0.12413	Lines substrate channel	α-357^Gly^
Phe-602	Phe, 1	No	Lines substrate channel	α-381^Phe^
Ala-603	Ala, 0.47988	Gly, 0.46717	Lines substrate channel	α-382^Ala^

**Fig. 2.**
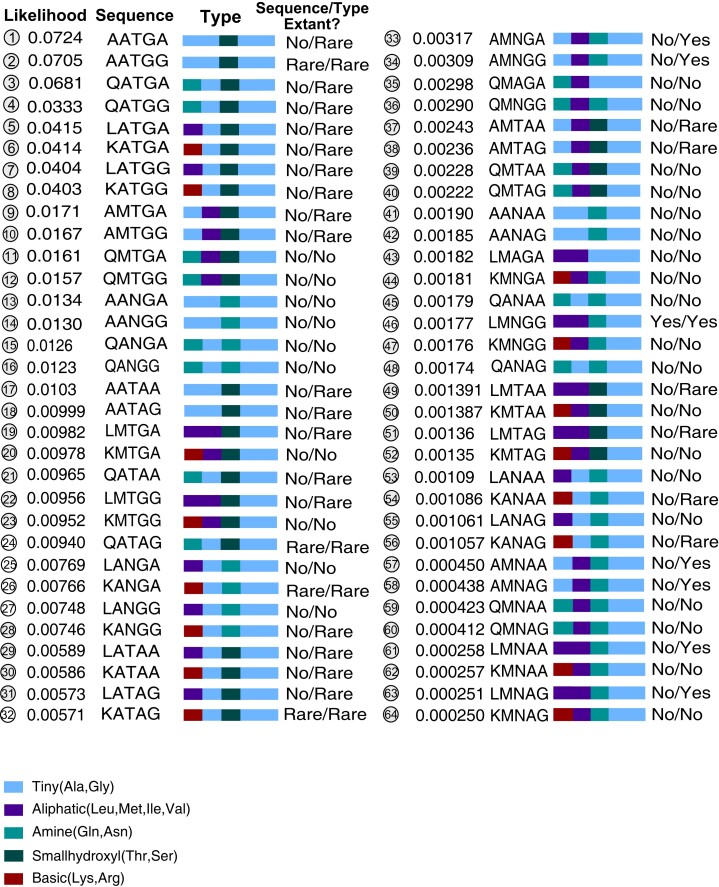
Ancestral sequence and state likelihoods. Likelihood ratios, amino acid sequence, and residue physicochemical states are shown for all combinations of the five variable sites (alignment sites 348, 495, 496, 576, and 603) predicted in the nitrogenase substrate channel ASR. “Rare” states occur in no more than two extant sequences.

Predicted combinations of residue states were evaluated in the ASR for the ancestor of Nif, Anf, and Vnf (fig. 1). The 64 evaluated ancestral sequence combinations of five variant sites had predicted joint likelihood ratios >0.00025% (fig. 2). Only 5/64 (7.8%) combinations were found in extant sequences (fig. 2). Of these five combinations, only one—the second-highest likelihood sequence, AATGG—was in the top 20 most-likely predicted ancestral combinations. Four of the five sequences were “rare,” observed in no more than two extant sequences; the only non-rare sequence (LMNGG) had the lowest likelihood of these five. Several sequence patterns inferred as high-likelihood reconstructed ancestors were not observed within extant nitrogenases. For example, in sampled extant sequences, Gln-348 does not co-occur with Gly-576; Ala-348 does not co-occur with Met-495; and Leu-348 does not co-occur with Ala-495. Ala-348 and Ala-495 only co-occur in one extant sequence.

It is possible that observed differences between extant sequences and inferred ancestors involve conservative amino acid substitutions that are unlikely to substantially affect substrate binding or phenotype. To test this, amino acids were recoded by general physiochemical properties, to identify radical changes of amino acid type. This showed that 29/64 (45%) of reconstructed ancestors contained substitutions yielding a combination not represented by extant sequences or physicochemical types (fig. 2). Of the 35 ancestors represented among extant physicochemical types, 28/35 (80%) were rare. The 32 most-likely ancestors all displayed physicochemical types that were rare or nonexistent within extant sequences. The top ten most-likely ancestors sampled rare physicochemical types only found within the divergent Nif/Anf/Vnf clade, not within Nif I/II.

**Fig. 3.**
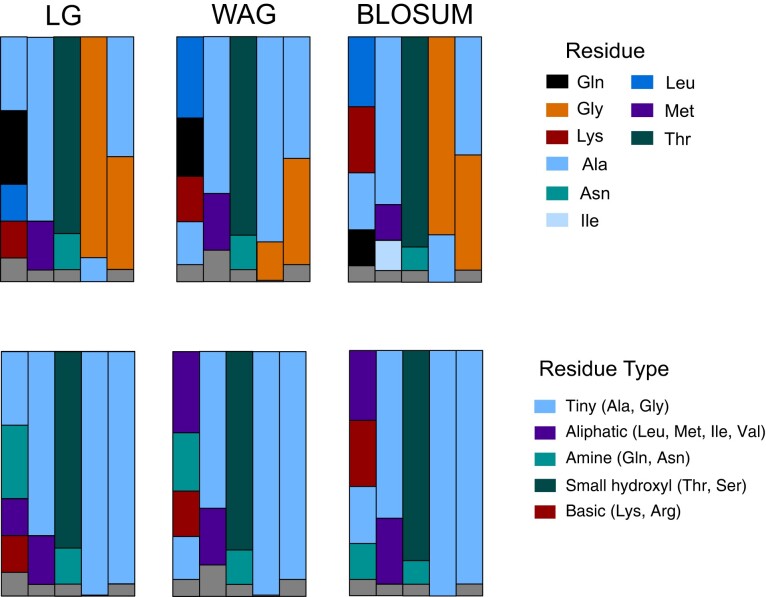
Substitution model effect on nitrogenase ASR. Proportional likelihoods are shown for residue states and physicochemical types at the five variable sites (alignment sites 348, 495, 496, 576, and 603) predicted in the nitrogenase ancestor for three different substitution models: LG + R9; WAG + R10; and BLOSUM62 + R10. Relative amino acid likelihoods for the nitrogenase ancestor were generally conserved across models at sites 495, 496, and 603. At site 576, LG and BLOSUM favor Gly over Ala (88% vs. 12% for LG and 81% vs. 19% for BLOSUM, respectively), whereas WAG favors Ala over Gly (88% vs. 12%, respectively). At site 348, all models recover a plurality of Ala, Glu, Gln, and Lys, with relative probabilities varying between 17.5% and 1.9%. Residue type combinations are robust to model selection across sites.

The effects of substitution models and topology on predicted ancestor sequence and type were also evaluated by repeating tree construction and ASR under WAG + R10 (the highest likelihood non-LG model) and BLOSUM62 + R10 (a lower likelihood, simple model). Substitution models slightly impacted recovered tree topologies within recently diverged lineages but did not alter phylogenetic relationships between major nitrogenase types and their outgroups. Relative likelihoods for specific amino acids are generally conserved at sites 495, 496, and 603, and sample the same plurality at site 576 (Gly, Ala) and site 348 (Ala, Glu, Gln, Lys) (fig. 3). Moreover, residue type combinations are robust to model selection.

## Discussion

These results suggest that the inferred ancestor of Nif, Vnf, and Anf nitrogenases most likely contained a substrate channel sequence distinct from that found in extant sequence space. Twenty-two of the 23 highest likelihood sequence combinations of the five variant substrate channel ancestral sites are not observed within known modern nitrogenases; the only represented sequence pattern occurs in only one observed extant enzyme, that of *Chloroflexales bacterium ZM16-3*. The top ten most-likely ASR residue combination physicochemical types were represented by very few extant sequences, and only within the divergent group of Nif that also contains the alternative nitrogenases. Divergent homologs within this clade may sample ancestral sequence patterns to a greater extent than type I or II nitrogenases. If so, characterizing these extant genes could provide useful analogs for ancestral phenotypes.

The conservative substitutions observed in the highest likelihood predicted ancestors are unlikely to radically influence substrate affinity or binding, at least when compared with the extant enzymes in the Nif/Anf/Vnf group. In the case of HCN, for example, because both modern Vnf (Seefeldt et al. 2013) and Nif can reduce this alternative substrate, there is no reason to believe that the ASR-predicted ancestors could not as well. Nevertheless, the observed departures from extant sequence space in many likely ancestor sequences open the possibility of greater phenotypic variation of substrate binding in nitrogenase ancestors. Recent work has indicated that even a small number of substitutions, even outside the active site, can elicit different ancestral substrate affinities (Tatsaki et al. 2021). This justifies experimental investigation of alternative substrate binding in reconstructed ancestor candidates, especially those displaying the novel residue type combinations sampled above.

Extant nitrogenases that remain unsampled and excluded from the tree could contain currently missing high-likelihood sequence combinations in the ASR. Additionally, likelihood ratios calculated from only a handful of sites do not represent overall sequence likelihoods, as site likelihoods are calculated independently. Thus, the most-likely ancestral site combinations in the substrate channel are unlikely to identify the sequence in the most-likely overall ancestor. However, the likelihood calculations for these substrate binding sites may be more informative for inferring ancestral phenotype changes than the overall ancestral sequence of the enzyme. Indeed, physicochemical type combinations predicted by the ASR are more robust to changes in substitution model and tree topology than sequence-level variation (fig. 3); this underscores the importance of considering physicochemical type for predicted substitutions. These results could be further contextualized by evaluating more ancient ancestors—for example, the last common ancestor of nitrogenases and maturases (Garcia et al. 2022)—and less-likely ancestral candidates with methods such as Bayesian sampling and laboratory reconstruction (Merkl and Sterner 2016; Garcia and Kaçar 2019; Selberg et al. 2021). But this likelihood-based, nitrogenase-exclusive approach provides the most conservative evaluation of functional hypotheses based on modern enzyme variants.

The results highlight the value of ASR as a first step in evaluating enzyme evolution hypotheses. In some cases, ASR itself may prove sufficient to refute certain evolutionary hypotheses; for example predicted ancestral states may be sterically incompatible with hypothesized substrate interactions or protein folding. Such results would preclude additional experimental work. It is also possible that when ASR fails to identify combinations of key ancestral residues absent from extant diversity, the null hypothesis of uniform phenotype across the reconstructed history should be preferred. However, even in this case, ASR will efficiently identify key sites to target for mutagenesis for in vitro or in vivo analyses—such as the novel sequence and type combinations sampled in this work.

## Materials and methods

An initial data set of extant nitrogenase D-subunit homologs was constructed from hits gathered using BLASTp to search NCBI's non-redundant protein database with a query sequence from *A. vinelandii* (WP_012698832.1). Hits were included for molybdenum (NifD), vanadium (VnfD), and iron-only (AnfD) nitrogenases and manually curated to remove partial sequences, prune oversampled clades, and ensure the presence of conserved sequence features known to be critical for N_2_ reduction (e.g., Cys-275, His-442). The curated initial data set included 385 nitrogenase sequences and 385 outgroup sequences from light-independent protochlorophyllide oxidoreductases (Boyd et al. 2011; Ghebreamlak and Mansoorabadi 2020). These initial homologs were aligned using MAFFT under auto-parameterization (selecting L-INS-i; Nakamura et al. 2018). Outgroup sequences were further subsampled to retain one sequence per phylum for major monophyletic groups (12 sequences). A second nested outgroup (five sequences) of Group IV-A nitrogenase-like homologs (*nfaD*; Raymond et al. 2004; North et al. 2020) was profile-aligned to the existing alignment with MAFFT, providing an additional ancestral node required to reconstruct the *nif/anf/vnf* ancestor as an internal node in the tree. The tree was rooted along the split between nitrogenases and the other oxidoreductases. Maximum-likelihood tree construction and subsequent ASR were performed in IQ-Tree (Nguyen et al. 2015), under the best-fit model identified by the Bayesian Information Criterion, LG + R9. Tree construction and ASR were also repeated using a similar, lower likelihood model, WAG + R10; and a simpler, lower likelihood model, BLOSUM62 + R10. Joint likelihoods were calculated as the product of individual residue likelihoods reported for sites of interest in the ASR state file. Amino acid states were grouped with the following physicochemical categories for residues: Acidic (Glu, Asp); Aliphatic (Ile, Leu, Met, Val); Amine (Gln, Asn); Aromatic (Trp, Tyr, Phe); Basic (Lys, Arg); Small Hydroxyl (Ser, Thr); Tiny (Ala, Gly).

## Data Availability

Data files used for analyses in this work are freely available at https://doi.org/10.6084/m9.figshare.c.6039527.
